# Self-generated peroxyacetic acid in phosphoric acid plus hydrogen peroxide pretreatment mediated lignocellulose deconstruction and delignification

**DOI:** 10.1186/s13068-021-02075-w

**Published:** 2021-11-25

**Authors:** Dong Tian, Yiyi Chen, Fei Shen, Maoyuan Luo, Mei Huang, Jinguang Hu, Yanzong Zhang, Shihuai Deng, Li Zhao

**Affiliations:** 1 Institute of Ecological and Environmental Sciences, Sichuan Agricultural University, Chengdu, 611130 Sichuan People’s Republic of China; 2 Department of Chemical and Petroleum Engineering, University of Calgary, 2500 University Dr. NW, Calgary, AB T2N 1N4 Canada; 3 College of Environmental Sciences, Sichuan Agricultural University, Chengdu, 611130 Sichuan People’s Republic of China

**Keywords:** Lignocellulose pretreatment, Peroxyacetic acid, Deconstruction mechanism

## Abstract

**Background:**

Peroxyacetic acid involved chemical pretreatment is effective in lignocellulose deconstruction and oxidation. However, these peroxyacetic acid are usually artificially added. Our previous work has shown that the newly developed PHP pretreatment (phosphoric acid plus hydrogen peroxide) is promising in lignocellulose biomass fractionation through an aggressive oxidation process, while the information about the synergistic effect between H_3_PO_4_ and H_2_O_2_ is quite lack, especially whether some strong oxidant intermediates is existed. In this work, we reported the PHP pretreatment system could self-generate peroxyacetic acid oxidant, which mediated the overall lignocellulose deconstruction, and hemicellulose/lignin degradation.

**Results:**

The PHP pretreatment profile on wheat straw and corn stalk were investigated. The pathways/mechanisms of peroxyacetic acid mediated-PHP pretreatment were elucidated through tracing the structural changes of each component. Results showed that hemicellulose was almost completely solubilized and removed, corresponding to about 87.0% cellulose recovery with high digestibility. Rather high degrees of delignification of 83.5% and 90.0% were achieved for wheat straw and corn stalk, respectively, with the aid of peroxyacetic acid oxidation. A clearly positive correlation was found between the concentration of peroxyacetic acid and the extent of lignocellulose deconstruction. Peroxyacetic acid was mainly self-generated through H_2_O_2_ oxidation of acetic acid that was produced from hemicellulose deacetylation and lignin degradation. The self-generated peroxyacetic acid then further contributed to lignocellulose deconstruction and delignification.

**Conclusions:**

The synergistic effect of H_3_PO_4_ and H_2_O_2_ in the PHP solvent system could efficiently deconstruct wheat straw and corn stalk lignocellulose through an oxidation-mediated process. The main function of H_3_PO_4_ was to deconstruct biomass recalcitrance and degrade hemicellulose through acid hydrolysis, while the function of H_2_O_2_ was to facilitate the formation of peroxyacetic acid. Peroxyacetic acid with stronger oxidation ability was generated through the reaction between H_2_O_2_ and acetic acid, which was released from xylan and lignin oxidation/degradation. This work elucidated the generation and function of peroxyacetic acid in the PHP pretreatment system, and also provide useful information to tailor peroxide-involved pretreatment routes, especially at acidic conditions.

**Graphical abstract:**

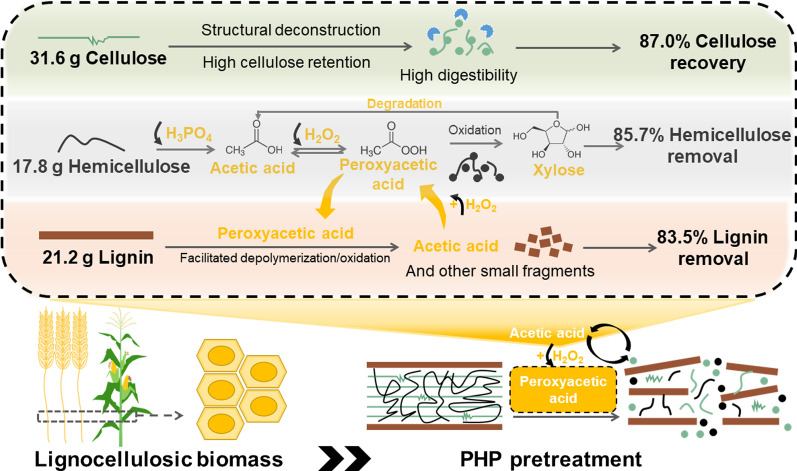

**Supplementary Information:**

The online version contains supplementary material available at 10.1186/s13068-021-02075-w.

## Background

The future limited availability of fossil fuels and skyrocketing global energy demand have been driving the rapid development of lignocellulosic biorefinery, which aims at processing biomass to bioenergy, materials and platform chemicals through chemical or biochemical routes [1–3]. Lignocellulose is a renewable and carbon–neutral bioresource that has received considerable attention over the past decades [4–6]. Although lignocellulose-based refineries are promising, the lignocellulose recalcitrance is known to hinder the efficient conversion [7–9]. Therefore, it is essential to disrupt the rigid structure using the pretreatment to enhance the usability of cellulose, hemicellulose, and lignin component for the subsequent downstream application [10].

In a multiproduct biorefinery concept, various pretreatment approaches, typically including physical (comminution, grinding and milling), chemical (alkali, acid, oxidizing agents, and organic solvents), biological, and a combination of these pretreatment techniques have been widely developed [6, 11]. Chemical pretreatment is the most promising method for lignocellulosic biomass [12]. Among various chemical methods, solvent-based pretreatments have been recognized to be effective in disrupting the hydrogen bonding network of the cellulosic component through the dissolution–regeneration mechanism [13]. Previous work has shown that the concentrated H_3_PO_4_ plus H_2_O_2_ (PHP) pretreatment exhibited good performance on removing hemicellulose and lignin fractions from lignocellulose biomass through an aggressive oxidation process while enhancing the subsequent enzymatic saccharification of cellulose component, even at high solids loadings [14]. Whereas, concentrated H_3_PO_4_ or H_2_O_2_ alone showed much lower ability in biomass deconstruction and delignification [15, 16]. The function and synergistic effect between H_3_PO_4_ and H_2_O_2_ are still unclear especially whether some strong oxidant intermediates have existed. Thus, it is necessary to acquire more detailed information about PHP pretreatment profile, which contributes to tailor technique routes for downstream valorizations of each fraction.

Our recent work has shown that PHP solvent system could degrade biomass lignin into low-molecular-weight fragments through C–O–C linkages cleavage and oxidative ring-opening reactions of lignin aromatics [13]. It was also shown the used H_3_PO_4_ could be recycled and reused through facile rotary evaporation for water removal. At the same time, the residual phosphorus in the wastewater could be removed using biochar adsorption to reduce phosphorus emissions [6]. At the optimized conditions, rather high cellulose hydrolysis and bioethanol conversion were achieved using simultaneous saccharification and fermentation process (SSF), e.g., 15.5 g ethanol was produced from 100 g wheat straw at rather high solid loadings [14, 17]. It has been evidenced the strong oxidation reaction that occurred in PHP pretreatment system played an important role in biomass deconstruction and delignification to ease cellulose hydrolysis [18]. However, the trigger of the oxidation process, types and working profile of the strong oxidant were still unclear.

It has been acknowledged that peroxy acids could produce hydroxonium (HO^+^) cations through heterolytic cleavage of the peroxidic bond (COO–OH). The generated strong electrophilic cations could react with the electron-rich sites in lignin, including both aromatic ring and olefinic side chain structures. Peroxy acids have been widely used for wood delignification, chemical pulps bleaching due to their strong oxidant ability, high selectivity and mild operating conditions [19, 20]. Among all of the peroxy acids, peroxyacetic acid could be operated at relatively low temperature with minor side reactions thus attracted the most research interest. Earlier work has shown that peroxyacetic acid solution could liquefy lignin component of liquid hot water treated biomass [21]. It was also shown peroxyacetic acid pretreatment could improve enzymatic digestibility of aspen wood. The resulting glucose yield was increased from 22 to 98% after peroxyacetic acid pretreatment [22]. However, these peroxyacetic acids were usually artificially added. If the peroxyacetic acid oxidant was in situ generated, the overall economy and sustainability of the lignocellulose biorefinery could be significantly enhanced.

Inspired by the well-researched peroxide-involved pretreatment that lignocellulose could be well deconstructed through the facile oxidation process either in acidic or alkaline conditions [23–25], this work assumed that peroxyacetic acid, an intermediate product that could be self-generated at acidic conditions, was responsible for the efficient delignification of PHP pretreatment. It was proposed that peroxyacetic acid was synthesized through H_2_O_2_ oxidation of acetic acid, which was released from biomass acetyl groups and lignin degradation. This work focused on determining the function of concentrated H_3_PO_4_ and H_2_O_2_ in the pretreatment system, and elucidating the mechanism of peroxyacetic acid-mediated deconstruction of lignocellulose. Wheat straw and corn stalk as representative agricultural residues were selected due to the characteristics of wide cultivation and abundance in agriculture. They were pretreated with various times, temperatures, and different H_3_PO_4_ to H_2_O_2_ ratios. Hemicellulose removal, delignification and cellulose retention of these two substrates after pretreatment were determined. To gain insights into the structural changes of the single component, microcrystalline cellulose, xylan and dealkalized lignin, alkali lignin, and cellulytic enzyme lignin were selected as cellulose, hemicellulose and lignin model compounds, respectively, and pretreated at the same conditions. In the PHP pretreatment system, the synthetic pathway of peroxyacetic acid and the degradation route of hemicellulose and lignin was proposed. This work provides insightful information for the oxidative deconstruction of lignocellulose, and also contributes to tailoring new peroxide-involved pretreatment systems especially in acidic conditions.

## Results and discussion

### Synergistic removal of hemicellulose and lignin in PHP pretreatment

Both hemicellulose and lignin were generally considered as physical hindrances that prevented the access of cellulase from biomass surface into cellulose interior during enzymatic hydrolysis process [26–28]. It was found that PHP pretreatment could remove most of the hemicellulose and lignin by relieving the hindrance of the barrier components, therefore, enriching the cellulose component [29]. The influence of H_3_PO_4_ to H_2_O_2_ ratios on hemicellulose and lignin removal could reflect the functions of H_3_PO_4_ and H_2_O_2_ in the pretreatment process.

**Fig. 1:**
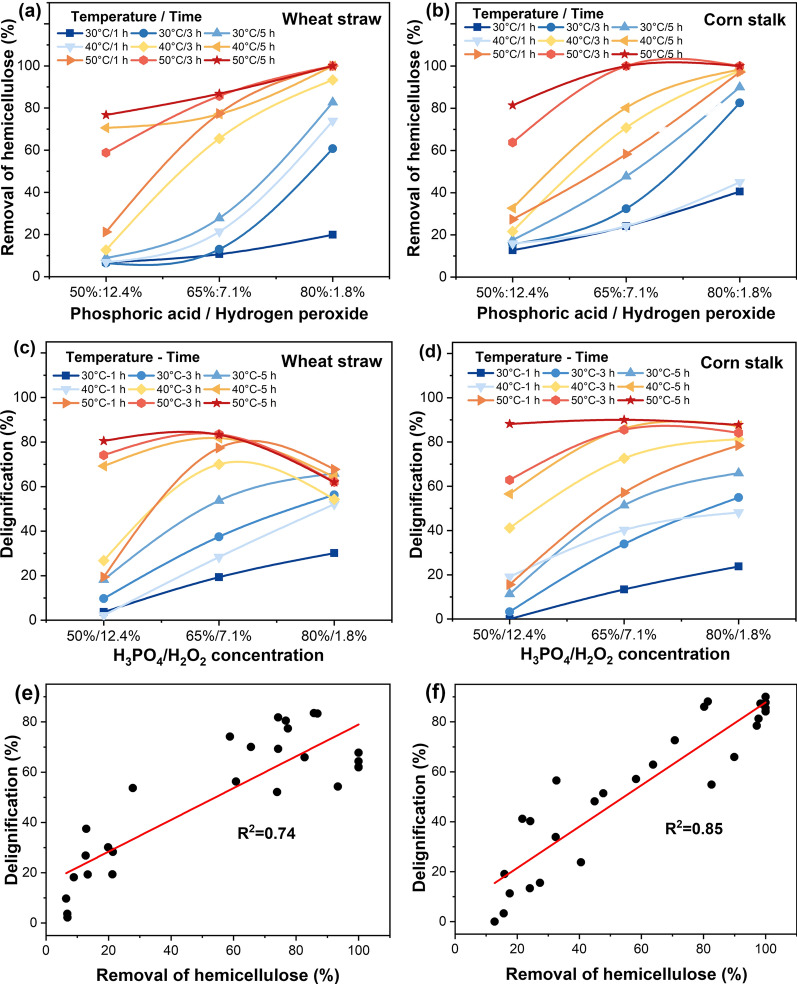
Effect of H_3_PO_4_/H_2_O_2_ concentrations on wheat straw and corn stalk hemicellulose removal and delignification. Pretreatment temperature, 30, 40, 50 °C, pretreatment time, 1, 3, 5 h. hemicellulose removal from wheat straw (**a**), hemicellulose removal from corn stalk (**b**), delignification from wheat straw (**c**), delignification from corn stalk (**d**). Correlations between delignification and hemicellulose removal of wheat straw (**e**) and corn stalk (**f**)

Hemicellulose has a branched structure and a low degree of polymerization and is more sensitive to degrade compared to cellulose component. Removal of substrate hemicellulose from wheat straw and corn stalk was varied with pretreatment intensities (Fig. 1a, b). Complete removal of hemicellulose components could be achieved for both substrates when extending pretreatment time or increasing cooking temperature. The hemicellulose removal increased with the increase of H_3_PO_4_ concentration for all the assessing time and temperature variables. It has been reported that the carbohydrates solubilization ability of concentrated H_3_PO_4_ and the strong deconstruction ability of PHP pretreatment contributed to the removal of hemicellulose [5, 30]. Therefore, hemicellulose removal effect was positively correlated with H_3_PO_4_ concentration, indicating that higher acid concentration increased the acidity of the pretreatment system to cause more hemicellulose degradation.

In addition, cellulose digestibility could be enhanced by the removal of lignin fraction due to the reduction of non-productive cellulase adsorption and increased cellulose accessible surface [31, 32]. As shown in Fig. 1c, d, after PHP pretreatment, all the assessing variables were influential in biomass delignification of these two substrates. When PHP pretreatment was conducted at mild conditions, i.e., short cooking time and low temperature, H_3_PO_4_ concentration was dominant in biomass delignification. Furthering enhancing the pretreatment severity significantly contributed to delignification. It was interesting that the optimized delignification of 83.5% and 90.0% for wheat straw and corn stalk, respectively, was achieved at the H_3_PO_4_ to H_2_O_2_ ratio of 65–7.1% instead of 80–1.8%.

Previous work has shown that acid-catalyzed lignin condensation would occur during prevalent single H_3_PO_4_ pretreatment, which limited the lignin extraction/degradation from lignocellulose [33, 34]. However, our recent work has shown that H_2_O_2_ addition could significantly limit lignin condensation reactions among aromatic units [13]. In the work reported here, it appeared that the deconstruction and oxidation function of H_2_O_2_ was obvious at rather severe pretreatment conditions. It was also shown that a better synergetic effect between H_3_PO_4_ and H_2_O_2_ occurred at higher temperatures. It could be interpreted that H_2_O_2_ underwent more protonation reactions to produce a large number of hydroxyl cations (HO^+^) under acidic conditions. The promoted electrophilic reactions likely eliminated the electron-rich active sites on the benzene rings to limit their condensation reactions with other electrophilic radicals involved in lignin depolymerization [19]. It was also likely that the severe pretreatment conditions significantly enhanced the overall oxidation ability of PHP pretreatment system thus facilitating hemicellulose and lignin removal through degradation reactions. It was also shown that H_3_PO_4_-catalyzed hemicellulose hydrolysis and H_2_O_2_-involved lignin oxidation were responsible for hemicellulose removal and delignification, respectively. When biomass delignification was plotted against its corresponding hemicellulose removal, a positive correlation of 0.74 and 0.85 was observed for wheat straw and corn stalk, respectively (Fig. 1e, f). This further supported the above analysis that a strong synergistic effect between H_2_O_2_ and H_3_PO_4_ existed during the PHP pretreatment process.

**Fig. 2:**
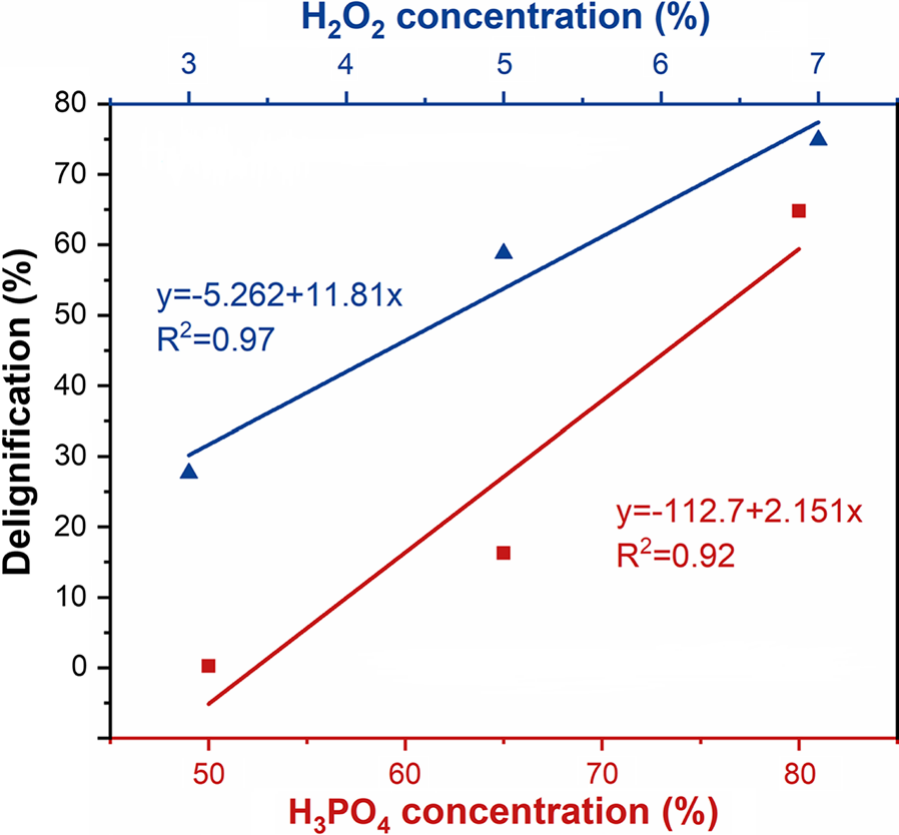
Influence of H_3_PO_4_ and H_2_O_2_ concentration on wheat straw delignification at 50 °C for 3 h. Blue line, when H_3_PO_4_ concentration was 65%, H_2_O_2_ concentration was designed as 3%, 5% and 7%. Red line, when H_2_O_2_ concentration was 1.8%, H_3_PO_4_ concentration was designed as 50%, 65%, and 80%

Previous work has shown that either H_3_PO_4_ or H_2_O_2_ alone was far less effective in biomass delignification and hemicellulose removal than their combination [16]. To further determine the functional role of H_3_PO_4_ and H_2_O_2_ on wheat straw delignification, various ratios of H_3_PO_4_ to H_2_O_2_ were further assessed for their biomass deconstruction profile at the optimized conditions (50 °C and 3 h). It was shown when H_3_PO_4_ concentration was 65%, increasing H_2_O_2_ concentration from 3.0 to 7.0% facilitated the delignification from 27.6 to 74.9% (Fig. 2). Similarly, when H_2_O_2_ concentration was 1.8%, increasing H_3_PO_4_ concentration from 50 to 80% also significantly enhanced wheat straw delignification from 0.3 to 64.8%. This indicated that the oxidative function of H_2_O_2_ needed the activation by H_3_PO_4_. It was clearly showed that the synergy between H_2_O_2_ and H_3_PO_4_ in biomass pretreatment. It appeared that H_3_PO_4_ could effectively disrupt biomass recalcitrant structure thus allow the H_2_O_2_ to access the inner of the substrate, while H_2_O_2_ was activated to generate strong oxidant to further facilitate biomass cell wall deconstruction [35, 36].

### Peroxyacetic acid-involved delignification profile of PHP pretreatment

To find the key factor that mediated the overall biomass deconstruction, the degradation products in the liquid fraction were analyzed using Gas chromatography–mass spectrometry (GC–MS). Six representative products, i.e., acetic acid, furfural, formic acid, furan, acrylic acid and benzoic acid were detected from the residue of PHP pretreatment (Additional file 1: Table S1). This result further demonstrated the strong oxidation ability of the PHP system thus various organic acids products were obtained after oxidation. Amongst these, the appearance of benzoic acid indicated that lignin could be further fragmented and degraded after acid-catalyzed β-O-4′ cleavage [37]. The presence of saturated and unsaturated aliphatic carboxylic acids, such as formic acid, acetic acid, and acrylic acid, showed more pieces of evidence for the deconstruction and oxidation of lignocellulose [38]. The appearance of furfural and furan suggested that pentose and hexose monosaccharides resulted from cellulose and hemicellulose hydrolysis underwent further degradation [39, 40].

**Fig. 3:**
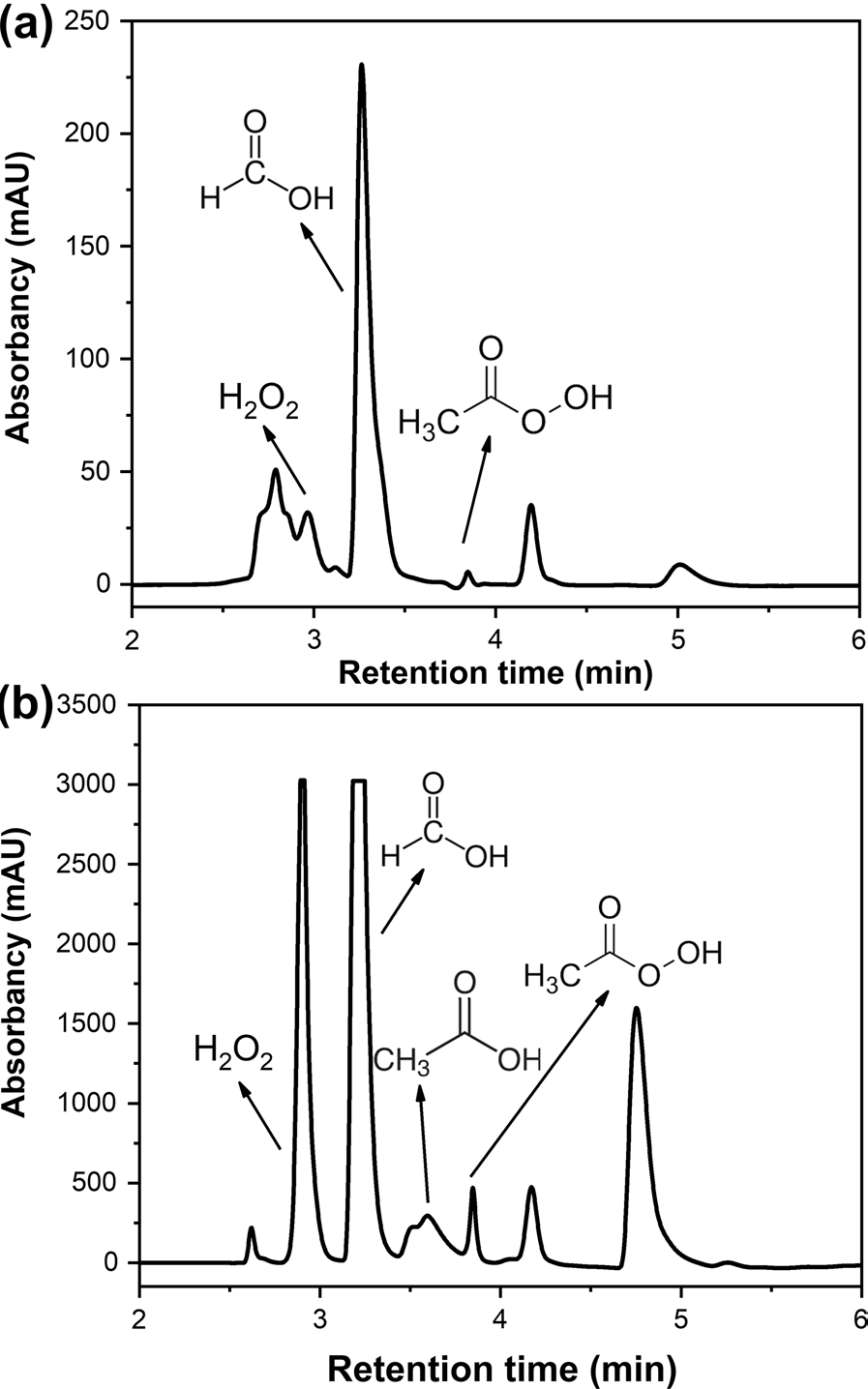
Determination of the main degradation products of PHP pretreatment through HPLC–UV, gas fraction of pretreatment after enrichment in water (**a**), the liquid fraction of the pretreatment after dilution in water (**b**)

To get more information about the degradation products of PHP pretreatment, the gas fraction generated during the cooking was simultaneously collected and analyzed using GC–MS (Additional file 1: Fig. S1). The spectrum results showed that a large peak correlated to carbon dioxide appeared, indicating that the benzene ring structure was oxidized to form quinone compounds, and even mineralized into carbon dioxide and water [41]. This clearly demonstrated the high extent of lignin oxidative degradation was corresponded to high delignification performance of PHP pretreatment (Fig. 1). Previous work has shown that when lignocellulose was pretreated under rather acidic conditions, much acetic acid and formic acid would be released through the deacetylation and oxidation process [5]. However, the GC–MS analysis showed that only a limited amount of acetic acid was detected in the volatile gas compounds (Additional file 1: Fig. S1, retention time, 6.38 min). The possible reason was that most of the generated acetic acid was consumed during the pretreatment or the concentration of acetic acid in the gas was close to detection limit. To quantify these degradation products, the gas fraction was enriched in water and analyzed using high performance liquid chromatography (HPLC). Interestingly, both formic acid and peroxyacetic acid were detected (Fig. 3a). Although the concentration of peroxyacetic acid was quite low even after enrichment, this result clearly demonstrated the existence of peroxyacetic acid during PHP pretreatment. This exciting result encouraged us to further look at the liquid fraction of PHP pretreatment. As shown in Fig. 3b, when the liquid fraction was detected after dilution using HPLC, representative products including H_2_O_2_, formic acid, acetic acid and peroxyacetic acid were clearly detected. Earlier work has shown that the acetic acid extraction was 0.21 ± 0.05% (w/w) at composition from 10 g dry biomass in pre-pulping hemicellulosic extraction hydrolysate [42]. However, due to the compositional complexity and structural unstability of these degraded products in the PHP pretreatment system, quantification of peroxyacetic acid has been technically challenging using HPLC detection. Since the peroxyacetic acid in the liquid fraction was responsible for the overall lignocellulose deconstruction performance, we roughly detected the content ratio of peroxyacetic acid in the liquid and gas fraction through chemical titration. The concentration of peroxyacetic acid in the liquid fraction was 5.4% (w/w), which was 100-fold higher than that in the gas fraction. The peroxyacetic acid concentration corresponded to 54 g/kg dried wheat straw substrate in optimized PHP pretreatment condition (50 °C, 3.0 h, and H_3_PO_4_/H_2_O_2_ loading of 65%/7.1%). It appeared that under such acidic conditions, acetic acid was oxidized by H_2_O_2_ thus a large amount of peroxyacetic acid was generated. It was also deduced the generated peroxyacetic acid with much higher oxidation ability acted as the key intermediate that influenced the overall lignocellulose deconstruction.

Previous work has shown that peroxyacetic acid would produce hydronium ion (HO^+^) in the reaction process to selectively oxidate lignin by replacing its side chains [19]. The guaiacyl and syringyl units were converted into quinone methide, and the aldehyde intermediate was converted into low molecular weight carboxylic acid by Baeyer–Villiger oxidation [43]. Therefore, it was speculated that the peroxyacetic acid formed during the PHP pretreatment reaction has a strong degrading effect on lignin.

**Fig. 4:**
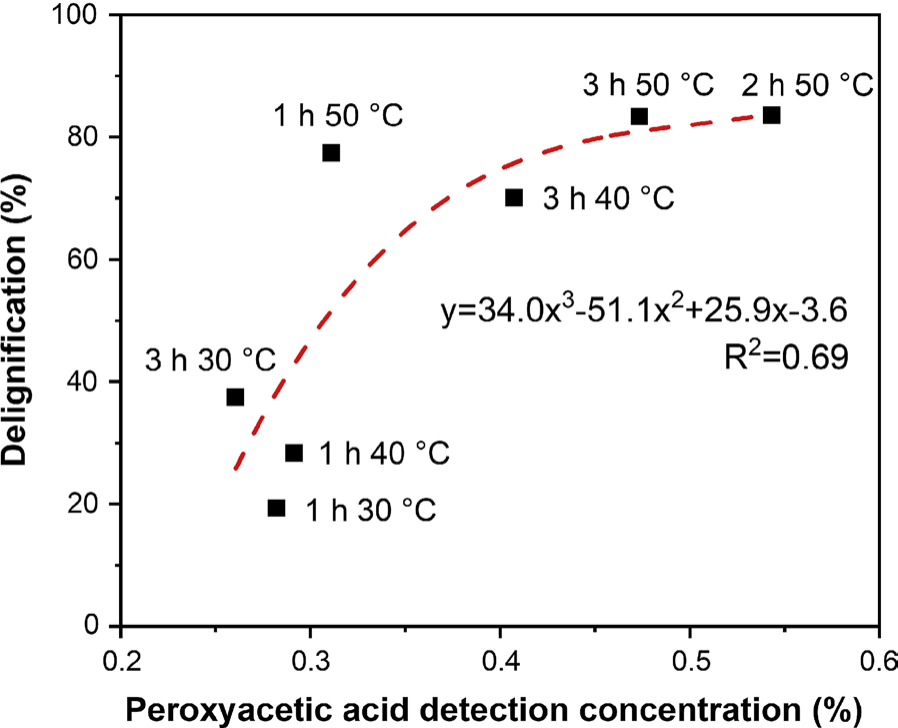
Positive non-linear correlation between delignification and peroxyacetic acid concentration

To explore whether there was a certain relationship between the delignification obtained by PHP pretreatment and the concentration of peroxyacetic acid formed by the pretreatment system, the content of peroxyacetic acid in the representative pretreatment conditions process was tested (Fig. 4). It was shown a positive non-linear correlation between delignification and peroxyacetic acid concentration was obtained. It appeared that the generated peroxyacetic acid might facilitate biomass delignification. However, it was also apparent that more peroxyacetic acid could be generated at severe pretreatment conditions. Both earlier work and the discussion above showed that increasing the pretreatment severity could enhance the delignification ability of PHP pretreatment. It was still conflicting that whether the generated peroxyacetic acid could boost biomass delignification.

**Fig. 5:**
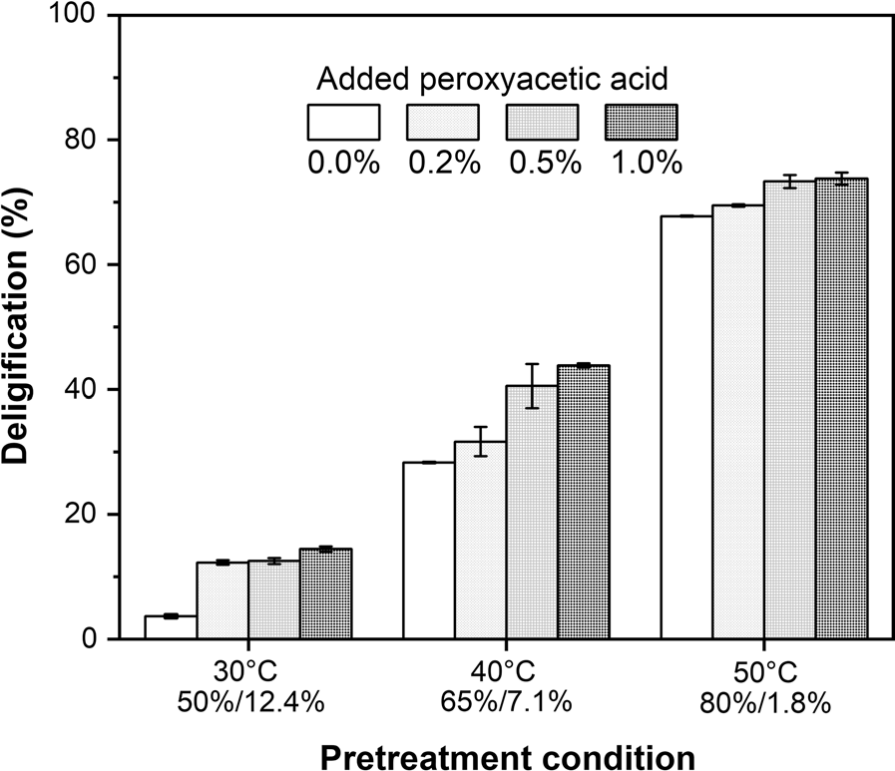
Influence of artificially added peroxyacetic acid (0.2, 0.5 and 1.0%) on wheat straw delignification. To minimize the interference of peroxyacetic acid self-generated in PHP solvent system, a short reaction time of 1 h was selected

To verify the boosting effect of peroxyacetic acid, as well as gain insights into the function of peroxyacetic acid in PHP pretreatment, an increasing amount of extra peroxyacetic acid with 0.2%, 0.5% and 1.0% concentration were artificially added to the PHP pretreatment system, respectively, to check whether the overall biomass deconstruction was enhanced (Fig. 5). Interestingly, compared to original PHP pretreatment systems, all these modified pretreatment systems gave increased biomass delignification. The extent of boosting effect was highly dependent on the additional amount of peroxyacetic acid and pretreatment temperature. It was shown the delignification boosting effect was obvious at mild conditions, corresponding to the maximum delignification enhancement of 14.4 and 43.81% at 30 °C and 40 °C, respectively, with 1% peroxyacetic acid addition. Despite the already rather high delignification of 67.8% at 50 °C, further adding peroxyacetic acid could still boost the delignification with the maximum enhancement to 73.8%. Moreover, biomass delignification was also enhanced with the increasing addition of peroxyacetic acid at the same temperature. These results clearly showed that peroxyacetic acid could facilitate biomass delignification during PHP pretreatment. Earlier work has shown peroxide-involved pretreatment showed a delignification fashion through strong ring-opening of guaiacyl and syringyl units [13]. The work reported here showed that the addition of peroxyacetic acid in PHP solvent system showed an obvious marginal effect on biomass delignification, likely due to decreasing content of guaiacyl and syringyl units in biomass.

### Formation pathway of peroxyacetic acid in PHP pretreatment system

**Fig. 6:**
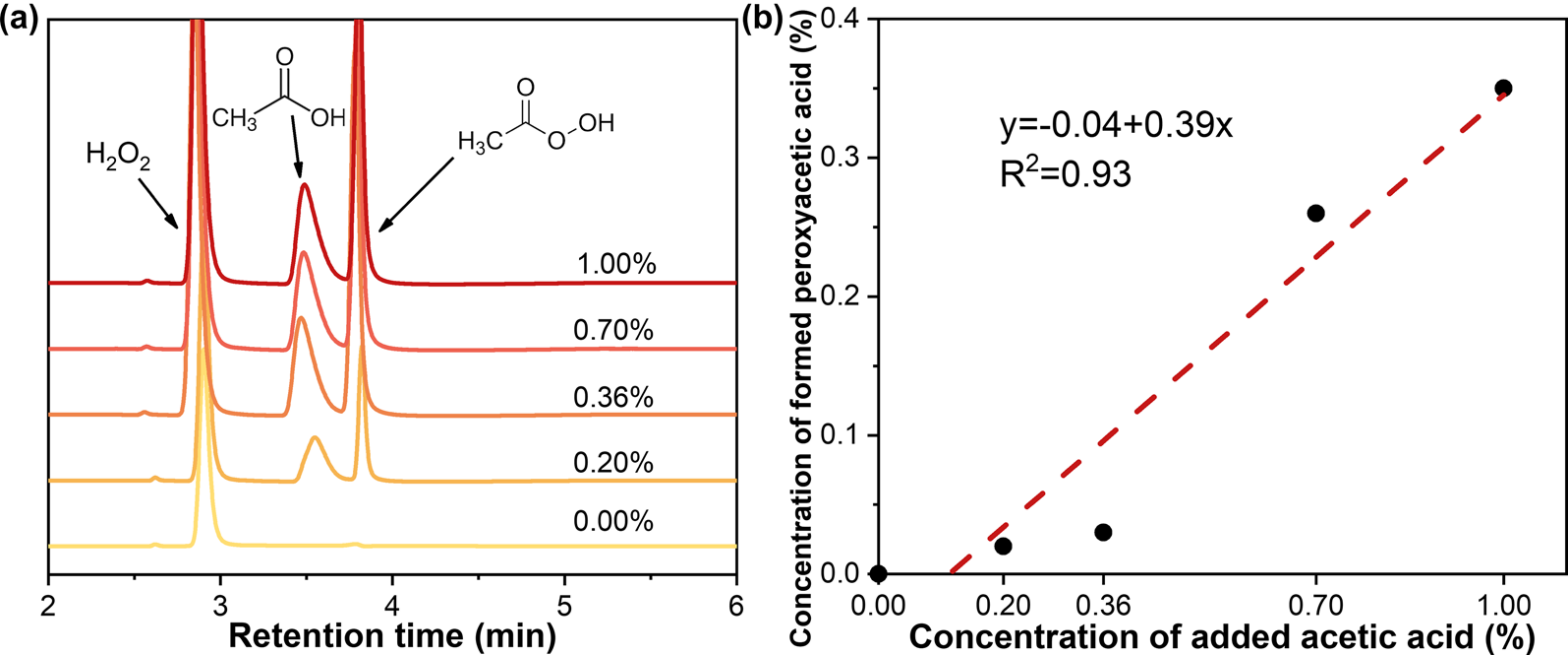
HPLC detection of peroxyacetic acid resulted from acetic acid addition in PHP system with an increasing concentration (**a**). The positive correlation between the concentration of peroxyacetic acid formed in the PHP system and the concentration of acetic acid artificially added, 0.00%, 0.20%, 0.36%, 0.70% and 1.00% (**b**)

Since peroxyacetic acid could be generated by an oxidation reaction between acetic acid and H_2_O_2_ under acidic conditions. It was also widely accepted that large amounts of acetic acid could be released during various chemical pretreatments [44, 45]. Therefore, it was proposed the peroxyacetic acid was generated through the oxidation of the released acetic acid and introduced H_2_O_2_ of PHP pretreatment. To verify this hypothesis, various amounts of acetic acid with 0.20, 0.36, 0.70, 1.00% loading was artificially added into the PHP solvent system without biomass involvement to check whether there was a correlation between peroxyacetic acid generation and acetic acid release. Since the pretreatment condition of H_3_PO_4_/H_2_O_2_ ratio, 65%/7.1%, with cooking temperature and time of 50 °C, 3 h showed good compromise of biomass delignification and polysaccharide yield, we selected it for the subsequent demonstration experiment. Earlier work has shown that about 1% acetic acid would be harvested in a typical pretreatment, thus we selected it as the maximum acetic acid addition accordingly. Results have shown that no peroxyacetic acid was detected in the original PHP solvent system (Fig. 6a), indicating PHP itself couldn’t produce peroxyacetic acid. However, increasing acetic acid addition significantly contributed to peroxyacetic acid generation (Fig. 6b). When the peroxyacetic acid generation was plotted against the acetic acid addition, an obvious positive linear correlation was obtained, which means that 1% (w/w) acetic acid addition could generate 0.35% (w/w) peroxyacetic acid with the excessive H_2_O_2_ in PHP liquor system. This analysis clearly supported the above hypothesis that the involvement of lignocellulose to release acetic acid was essential for peroxyacetic acid formation. Nonetheless, the specific source of acetic acid during PHP pretreatment, the formation route of peroxyacetic acid and the deconstruction pathway of lignocellulose components need to be further studied.

To address the above concerns, the structural changes of representative biomass model compounds during PHP pretreatment at the same conditions were traced. Microcrystalline cellulose was selected as cellulose model in this case due to its high purity and small particle size. After pretreatment, both acetic acid and peroxyacetic acid were not detected in the liquid fraction. This indicated that cellulose or its hydrolyzed products hardly participated in the formation of acetic acid. In PHP pretreatment, cellulose was deconstructed or swelled by concentrated H_3_PO_4_ and then regenerated after water dilution. The above analysis showed rather high cellulose yield was obtained after PHP pretreatment, which was in line with the results here that only a few parts of cellulose were degraded.

**Fig. 7:**
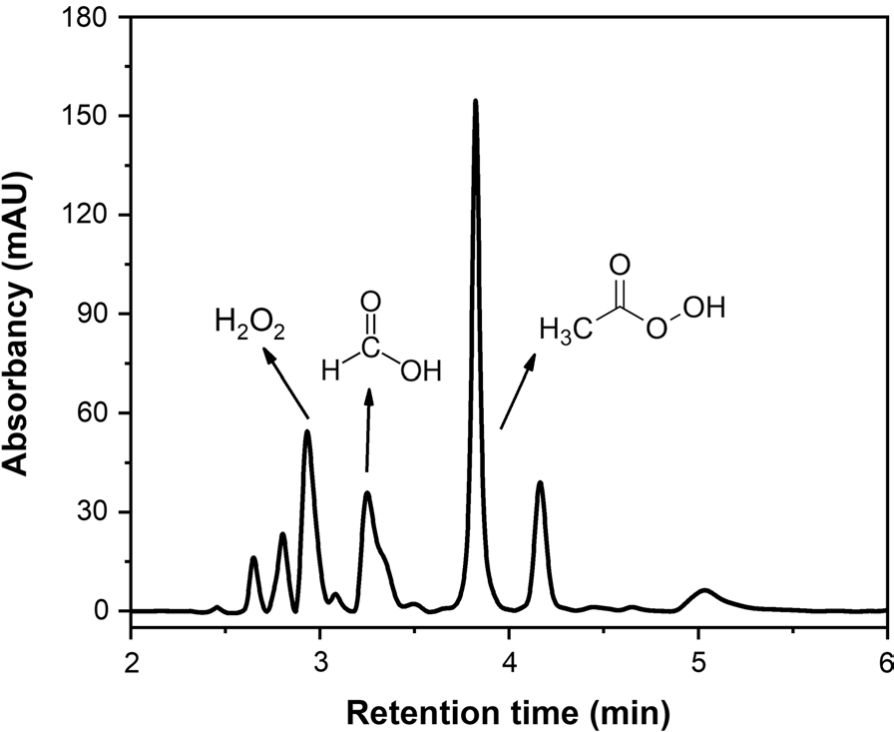
Determination of the main compounds in liquid fraction after PHP pretreatment of xylan through HPLC–UV

The major composition of the entire hemicellulose presented in agricultural residues was dominantly represented by xylan. When xylan was used as the hemicellulose model compound for the assessment, a variety of deconstruction products in both gas and liquid fraction were detected by GC–MS and HPLC–UV, respectively. It was shown acetic acid was detected in the gas fraction, while peroxyacetic acid was also traced in the liquid fraction (Fig. 7). To further verify the existence of acetic acid in the liquid fraction, HPLC-RI with higher resolution was complementally conducted (Additional file 1: Figs. S2, S3, S4). Results showed a trace amount of acetic acid was detected. These results clearly showed that acetic acid could be released under PHP condition, which was rapidly in situ converted into peroxyacetic acid with the oxidation of excessive H_2_O_2_ thus limited acetic acid was detected in the liquid fraction. In nature, the C-2 and C-3 positions of linear xylan are replaced by arabinose and acetyl groups, which could be hydrolyzed at acid environment to release acetic acid. It appeared that under acidic PHP environment, xylan was deconstructed and hydrolyzed rapidly to release acetic acid. In addition to acetic acid, formic acid was also simultaneously detected (Fig. 7). The appearance of formic acid indicated that xylan underwent both acid hydrolysis and further deconstruction reactions. It appeared that hexose in xylan was dehydrated to form 5-methyl furfural, and further completely deconstructed to levulinic acid and formic acid under severe conditions [46].

**Fig. 8:**
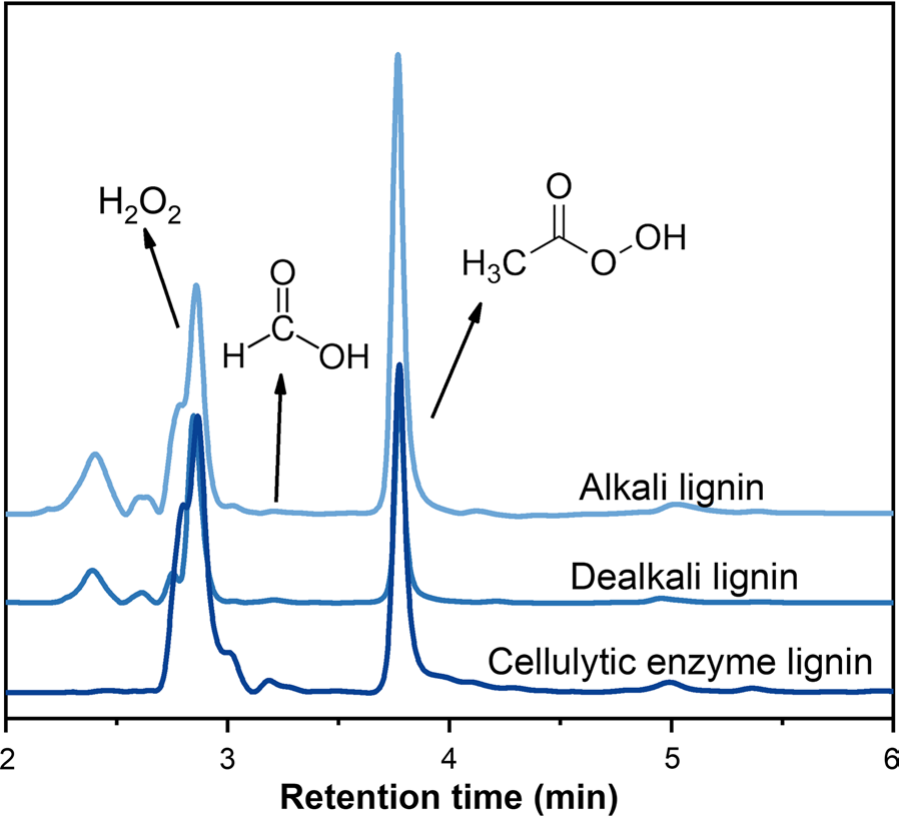
Determination of the main compounds in the liquid fraction of alkali, dealkali and cellulytic enzyme lignin after PHP pretreatment through HPLC–UV

When alkali, dealkali and cellulytic enzyme lignin were selected as lignin model compounds, the deconstruction products in the liquid fraction were assessed using HPLC–UV (Fig. 8). It was shown peroxyacetic acid was quite dominant among these three liquid fractions. These results also demonstrated that peroxyacetic acid could be produced from the degradation and oxidation of lignin, even though lignin types were varied. When these gas fractions were further identified by GC–MS, various degradation products including acetic acid were detected (Additional file 1:Table S2). It appeared that alkali lignin tended to give more kinds of degradation products compared to dealkali and cellulytic enzyme lignin. It was also shown that apart from the acetic acid generation that was involved for the subsequent peroxyacetic acid formation, small molecular formic acid, saturated fatty acids with longer molecular chains, such as *n*-undecanoic acid and *n*-dodecanoic acid, unsaturated fatty acids, such as acrylic acid and crotonic acid, aromatic acids, and a large amount of low molecular esters were also produced. These results clearly showed the degradation route of lignin in PHP pretreatment. Peroxyacetic acid exhibited an aggressive oxidative degradation effect on biomass lignin in the PHP pretreatment system. After the lignin was degraded into various small molecular compounds, most of the degradation products were removed from the system through the subsequent washing stage. Therefore, high delignification was achieved with the mediation of peroxyacetic acid.

**Fig. 9:**
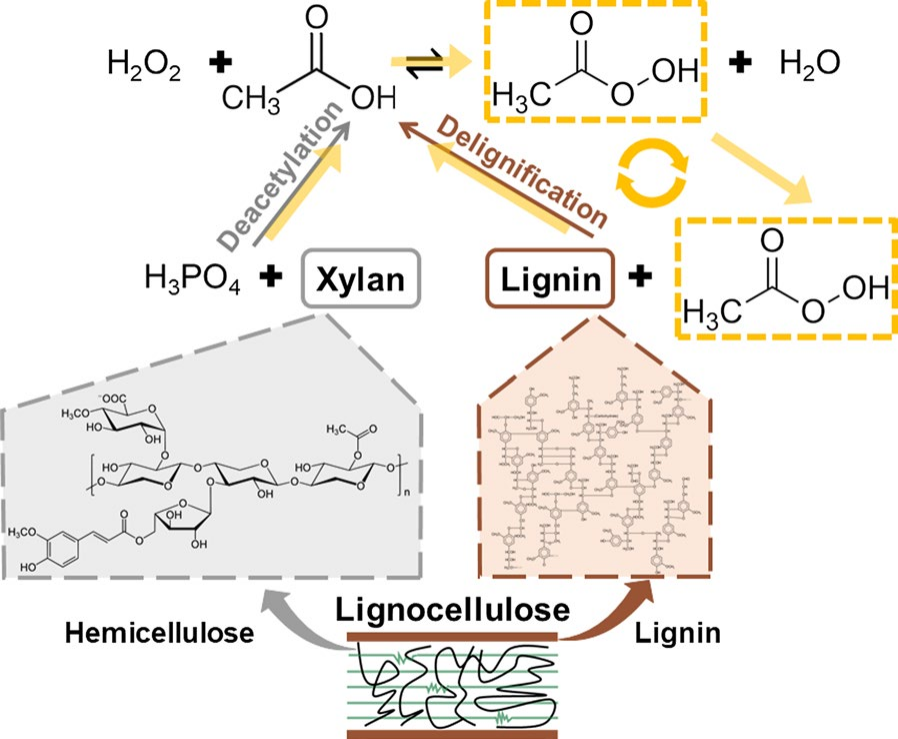
Formation pathway of peroxyacetic acid in PHP pretreatment system. The yellow arrow indicates the continuous production of peroxyacetic acid enhanced the synergistic effect of PHP pretreatment

**Fig. 10:**
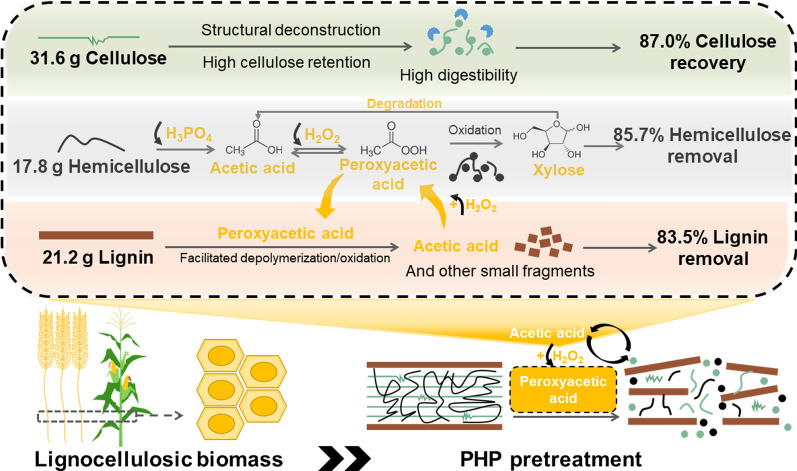
Mechanism of self-generated peroxyacetic acid in PHP pretreatment mediated lignocellulose deconstruction

According to the discussion above, the profile of PHP pretreatment, route of peroxyacetic acid generation (Fig. 9) and delignification mechanism (Fig. 10) were proposed. In the initial stage of PHP pretreatment, lignocellulose was penetrated by the concentrated H_3_PO_4_, while hemicellulose underwent acid-catalyzed hydrolysis and dehydration due to its branched and amorphous chemical structure [47]. The acetyl groups on the branches of hemicellulose structure released and formed acetic acid. Then H_2_O_2_ in PHP solvent system reacted with acetic acid in acidic environment to form peroxyacetic acid, which initiated the selective lignin oxidation and degradation. Peroxyacetic acid then oxidized xylose and lignin to produce more acetic acid [48], which consequently boosted the peroxyacetic acid formation and biomass deconstruction. Due to the continuous production of peroxyacetic acid, the cyclic synergistic effect of PHP pretreatment was continuously increasing. The lignin component with rather high structural recalcitrance underwent both fragmentation and oxidation-induced ring-opening reactions. These products contained low-molecular-weight monocarboxylic acids, saturated long-chain aliphatic carboxylic acids, unsaturated short-chain fatty acids, and some aromatic compounds. These small molecular compounds were removed from the substrate along with the washing process after the pretreatment. These acidic and oxidative conditions also showed a rather high ability to deconstruct cellulose, corresponding to about 87.0% recovery with high digestibility [49]. The formation of peroxyacetic acid significantly boosted lignin degradation to enhance the overall deconstruction effect of lignocellulose. The boosting effect of the self-generated peroxyacetic acid thus could mediate the synergy between hemicellulose removal and biomass delignification to achieve a better deconstruction effect.

## Conclusion

The synergistic effect of H_3_PO_4_ and H_2_O_2_ in the PHP solvent system could efficiently deconstruct wheat straw and corn stalk lignocellulose through an oxidation-mediated process. The main function of H_3_PO_4_ was to deconstruct biomass recalcitrance, degrade hemicellulose through acid hydrolysis, while the function of H_2_O_2_ was to participate in the formation of peroxyacetic acid. Peroxyacetic acid with higher oxidation ability was generated through the reaction between H_2_O_2_ and acetic acid, which was released from xylan and lignin oxidation/degradation. This work showed insights into the newly developed PHP pretreatment on its high efficiency of lignocellulose deconstruction, and also provide useful information to tailor peroxide-involved pretreatment routes, especially at acidic conditions.

## Experimental

### Materials

Wheat straw and corn stalk were collected from the farm of Sichuan Agricultural University (Chengdu, China), air-dried and ground by a high-speed grinder, screened to the particle sizes of 40 mesh (i.e., 0.45 mm). The unpretreated feedstocks, pretreated wheat straw, pretreated corn stalk, and the obtained lignin main chemical compositions were tested according to the Klason procedure (standard method Tappi Standard T222 om-88) [16, 50]. The main chemical composition of wheat straw and corn stalk were as follows, wheat straw, cellulose, 31.6 ± 0.75%, hemicellulose, 17.8 ± 0.40%, lignin, 21.2 ± 0.19%, ash, 7.8 ± 0.45%, extractives, 11.3 ± 0.69%. Corn stalk, cellulose, 35.8 ± 0.45%, hemicellulose, 19.2 ± 0.30%, lignin, 23.2 ± 0.36%, ash, 6.7 ± 0.60%, extractives, 4.1 ± 0.66%. Microcrystalline cellulose and xylan were purchased from Sigma-Aldrich (Beijing, China). The de-alkaline lignin and alkali lignin were purchased from J&K Scientific Ltd., China. Other reagents and solvents were purchased from Kelong Chemical Regent Co., Ltd. (Chengdu, China) and used as received.

### PHP pretreatment of lignocellulose

PHP pretreatment was carried out in a 250 mL Schott Duran with 10.0 g straw (dry basis) and 100.0 g PHP mixture. The sealed bottles were shaken in an incubator with 180 rpm for pretreatment. After the reaction, pretreatment was ceased by dilution using fivefolds distilled water. The solid fraction was washed until pH neutral, while the liquid fraction was collected for further use. The washed substrates were stored in a freezer at − 20 °C for further analysis. To collect and analyze gas products of PHP pretreatment, a tube was connected to the Schott Duran with a safety bottle (Additional file 1: Fig. S5). The collection bottle was filled with water before the reaction. Pretreatment temperature and time were investigated in the range of 30–50 °C and 1.0–5.0 h, respectively. The H_3_PO_4_ proportions of 50–80% (w/w) in the PHP mixture were also investigated with the corresponding H_2_O_2_ proportion of 12.4–1.8% (w/w).

Hydrolysate from the Klason analysis was analyzed for acid-soluble lignin by a UV spectrophotometer at 205 nm. The determined content of glucan, xylan, acid-soluble and insoluble lignin in hydrolysate represented for cellulose, hemicellulose and lignin content, respectively. The glucan and xylan were measured by high-performance liquid chromatography (HPLC) (Flexar, PerkinElmer, Inc., Waltham, MA, USA). The HPLC is equipped with an SH1011 column (Shodex, Showa Denko America, Inc., New York, NY, USA). The mobile phase was 0.05 mol/L H_2_SO_4_ at a flow rate of 0.8 mL/min, column and detector were operated at 60 °C and 50 °C, respectively. Cellulose retention was calculated as the cellulose weight after pretreatment divided by the total cellulose content in the starting substrate. Lignin or hemicellulose removal was calculated as the lignin or hemicellulose loss during the pretreatment divided by the total lignin or hemicellulose content in the starting substrate.

### PHP pretreatment of lignocellulose model compounds

The optimized PHP pretreatment conditions (1:10 solid–liquid ratio) were employed for model compounds pretreatment. After the reaction, a certain amount of the reaction product was qualitatively analyzed by GC–MS and HPLC. The sample detection procedure was the same as those for wheat straw and corn stalk. In addition, To elucidate the delignification in PHP system with different ratios of H_3_PO_4_ and H_2_O_2_, cellulolytic enzyme lignin (CEL) was isolated from wheat straw as a comparison. CEL was extracted with toluene/ethanol (2:1, v/v) in a Soxhlet instrument for 8 h. Afterwards, the extracted wheat straw was ball-milled and the subsequent lignin extraction was conducted according to the methods described previously [51, 52]. The detailed experimental flowchart of CEL was shown in Additional file 1: Fig. S6.

### Characterization of substrates and deconstructed products

Gas samples were analyzed using gas chromatography–mass spectroscopy analysis (Clarus SQ 8T GC/Mass Spectrometer, PerkinElmer) after pretreatment. The initial temperature was remained at 70 °C for 3 min and then increased to 220 °C with a heating rate of 20 °C/min. The heating was stabilized at 220 °C for 15 min. Helium was the carrier gas at a flow rate of 1 mL/min with a split ratio of 20:1. The ion source temperature was 230 °C, and the DBFFAP capillary column was 30.0 m × 250 μm. The reaction product was determined by HPLC equipped with a column (RP-18, PerkinElmer, Inc., Waltham, MA, USA) [53]. The UV detector was set at 205 nm. 15 mmol/L acetonitrile/potassium dihydrogen phosphate buffer (18/82, v/v) at pH 2–2.5 was employed as a mobile phase with a flow rate of 0.8 mL/min. The temperature of the column oven was set at 25 °C. 10 μL of the prepared sample with a concentration of 5.0 mg/mL was injected into the HPLC system for analysis. The peroxyacetic acid concentration was measured with Chinese standard GB/T 19104–2008. 50 mL high purity water, 5 mL sulfuric acid solution and three drops of manganese sulfate solution were added to the iodine flask, the mixed solution was cooled down at 4 °C. Peroxyacetic acid solution was transfered into the cooled iodine flask, then calibrated potassium permanganate solution was added. The mixed solution system became light pink color. 1 mL of potassium iodide solution and three drops of ammonium molybdate solution was subsequently added. The mixed solution was shaken well and placed in dark for 8 min. The calibrated sodium thiosulfate solution was used to titrate until the color was light yellow, then 1 mL starch indicator was added, and the mixed solution was sequentially titrated. The volume of titrimetric sodium thiosulfate solution was recorded and calculated through the following equation.

$$X=\frac{\left[{C}\left ({{\mathrm{Na}}_{2}{\mathrm{S}}_{2}{\mathrm{O}}_{3}}\right ) \times {V}\left ({{\mathrm{Na}}_{2}{\mathrm{S}}_{2}{\mathrm{O}}_{3}} \right ) \times {M}\left ({{\mathrm{CH}}_{3}\mathrm{COOH}} \right )\right]}{2m}\times 100\%$$

where *X* is the mass fraction of peroxyacetic acid (%), *C* is the concentration of sodium thiosulfate solution (mol/L), *V* is the volume of sodium thiosulfate solution consumed by titration (mL), *M* is the molar mass of peroxyacetic acid (g/mol), *m* is the weight of peroxyacetic acid solution (g).

## Supplementary Information

**Additional file 1: Table S1.** The main components of the pretreated-biomass deconstruction product (via GC–MS). **Figure S1.** Determination of the gas products of wheat straw after PHP pretreatment through GC–MS. **Figure S2.** Determination of the products of xylan after PHP pretreatment through HPLC-RI. **Figure S3.** The enlarged identified peak of acetic acid at the retention time of 16.00 min. **Figure S4.** The determination of the main compounds in liquid fraction after PHP pretreatment of xylan, alkali, dealkali and cellulolytic enzyme lignin through HPLC-RI. **Table S2.** Determination of the main degradation products of lignin model after PHP pretreatment through GC–MS. **Figure S5.** Device for collecting gas generated by PHP pretreatment. **Figure S6.** Isolation procedure for cellulolytic enzyme lignin.

## Data Availability

All the data analyzed during this study have been included in this article.

## Abbreviations

PHP: Phosphoric acid plus hydrogen peroxide
SSF: Simultaneous saccharification and fermentation
GC–MS: Gas chromatography–mass spectrometry
HPLC: High performance liquid chromatography
UV: Ultraviolet light detector
RI: Refractive index detectors
CEL: Cellulolytic enzyme lignin
