# Supplementary material for: Self-generated peroxyacetic acid in phosphoric acid plus hydrogen peroxide pretreatment mediated lignocellulose deconstruction and delignification
Source: Biotechnol Biofuels. 2021 Nov 25;14:224. doi: 10.1186/s13068-021-02075-w (PMC8614055; doi:10.1186/s13068-021-02075-w)
Supplement: Supplementary file 1 — Additional file 1: Table S1. The main components of the pretreated-biomass deconstruction product (via GC–MS). Figure S1. Determination of the gas products of wheat straw after PHP pretreatment through GC–MS. Figure S2. Determination of the products of xylan after PHP pretreatment through HPLC-RI. Figure S3. The enlarged identified peak of acetic acid at the retention time of 16.00 min. Figure S4. The determination of the main compounds in liquid fraction after PHP pretreatment of xylan, alkali, dealkali and cellulolytic enzyme lignin through HPLC-RI. Table S2. Determination of the main degradation products of lignin model after PHP pretreatment through GC–MS. Figure S5. Device for collecting gas generated by PHP pretreatment. Figure S6. Isolation procedure for cellulolytic enzyme lignin. [file 13068_2021_2075_MOESM1_ESM.docx]

Electronic Supplementary Information for

**Self-generated peroxyacetic acid in phosphoric acid plus hydrogen peroxide pretreatment mediated lignocellulose deconstruction and delignification**

Dong Tian^1^, Yiyi Chen^1^, Fei Shen^1^*, Maoyuan Luo^1^, Mei Huang^1^, Jinguang Hu^2^, Yanzong Zhang^3^, Shihuai Deng^3^ and Li Zhao^1^*

^1^Institute of Ecological and Environmental Sciences, Sichuan Agricultural University, Chengdu, Sichuan 611130, PR China

^2^Department of Chemical and Petroleum Engineering, University of Calgary, 2500 University Dr. NW, Calgary, AB T2N 1N4, Canada

^3^College of Environmental Sciences, Sichuan Agricultural University, Chengdu, Sichuan 611130, PR China

*Corresponding authors:

Fei Shen: fishen@sicau.edu.cn

Li Zhao: zhaoli@sicau.edu.cn

Contents

Ten pages (including cover sheet)

Two table (Table S1-2)

Six figures (Figure S1–6)

**Table S1** The main components of the pretreated-biomass deconstruction product (via GC-MS).

| Deconstruction products | Time (min) |
| --- | --- |
| Acetic acid | 6.60 |
| Furfural | 6.94 |
| Formic acid | 7.14 |
| Furan | 8.24 |
| Acrylic acid | 8.44 |
| Benzoic acid | 12.13 |


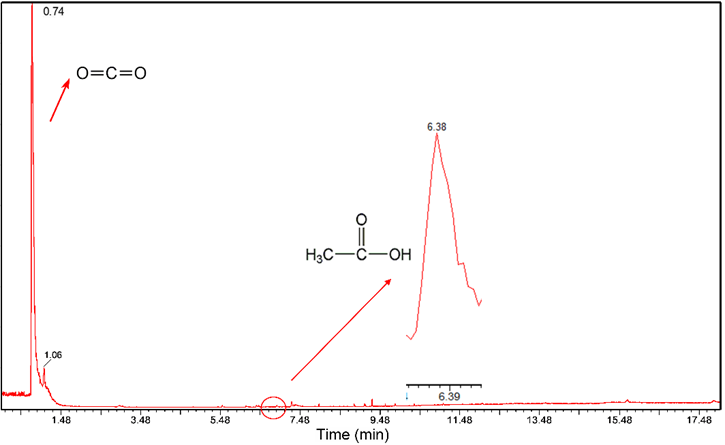


**Figure S1** Determination of the gas products of wheat straw after PHP pretreatment through GC-MS. The identified peaks, 0.74 min: Carbon dioxide, 6.38 min: Acetic acid.


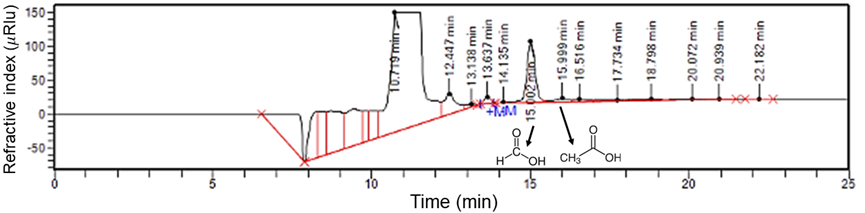


**Figure S2** Determination of the products of xylan after PHP pretreatment through HPLC-RI. The identified peaks, 15.00 min: Formic acid, 16.00 min: Acetic acid.


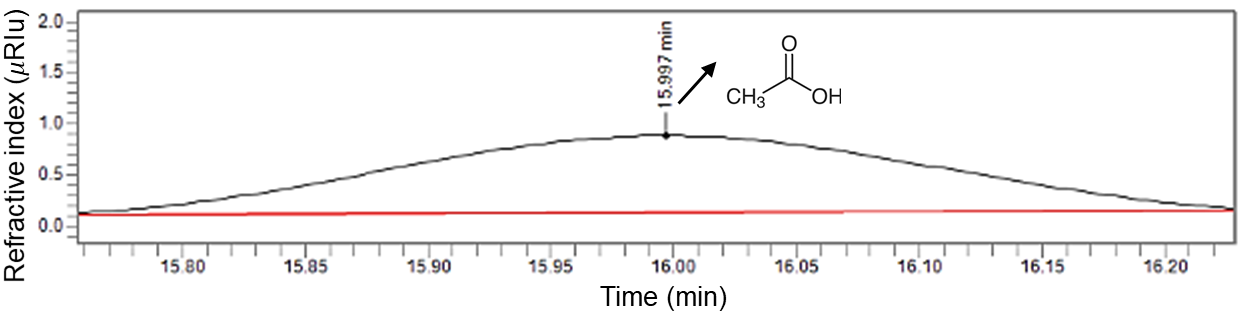


**Figure S3** The enlarged identified peak of acetic acid at the retention time of 16.00 min.


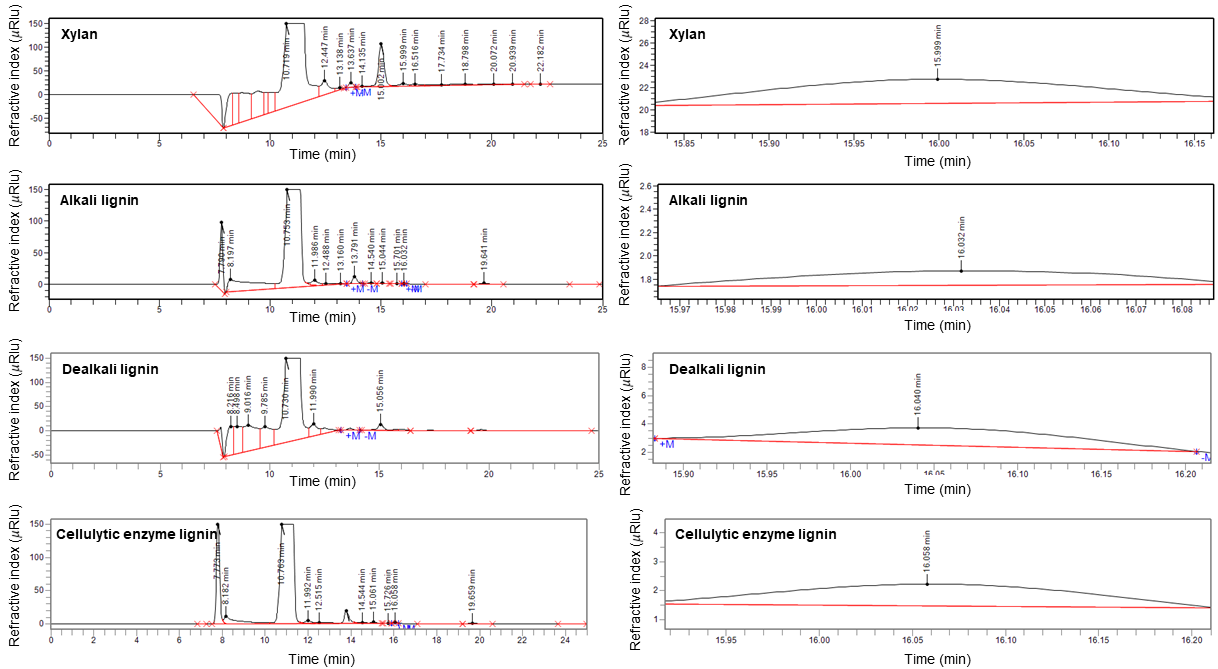


**Figure S4** The determination of the main compounds in liquid fraction after PHP pretreatment of xylan, alkali, dealkali and cellulolytic enzyme lignin through HPLC-RI, the right four figures are partial detection enlargement of acetic acid to the left four figures for a closer look at the peak intensity.

**Table S2** Determination of the main degradation products of lignin model after PHP pretreatment through GC-MS.

| PHP pretreatment | | |
| --- | --- | --- |
| Alkali lignin | Lignin (dealkalized) | Enzymatic hydrolysis lignin |
| 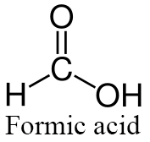 | 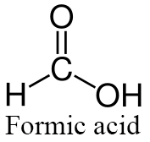 | 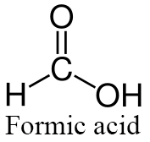 |
| 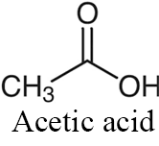 | 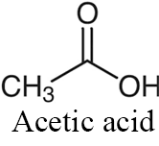 | 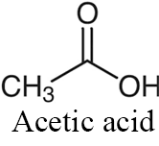 |
| 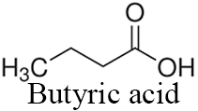 | 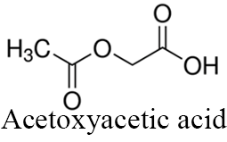 | 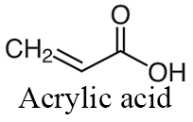 |
| 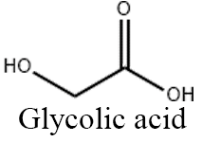 | 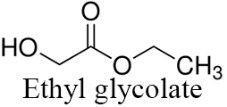 | 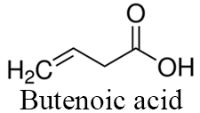 |
| 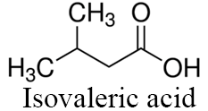 | 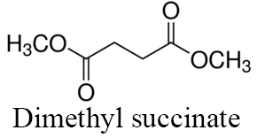 | 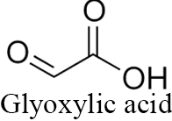 |
| 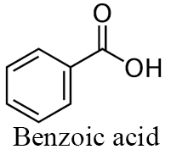 | 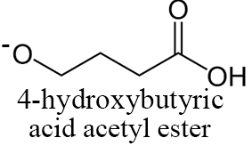 | 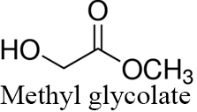 |
| 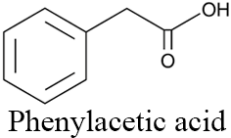 | 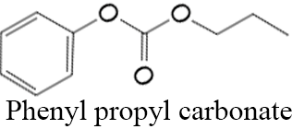 | 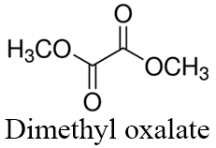 |
| 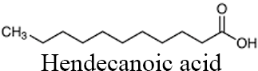 | - | 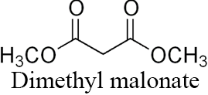 |
| 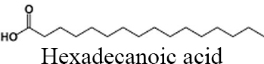 | - | - |
| 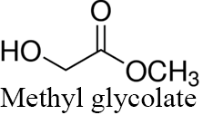 | - | - |
| 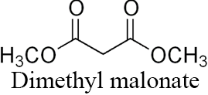 | - | - |
| 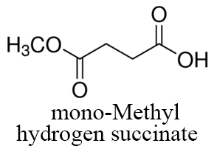 | - | - |
| 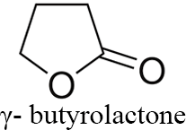 | - | - |
| 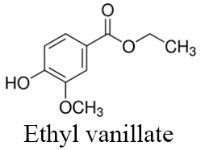 | - | - |
| 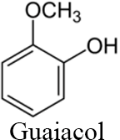 | - | - |


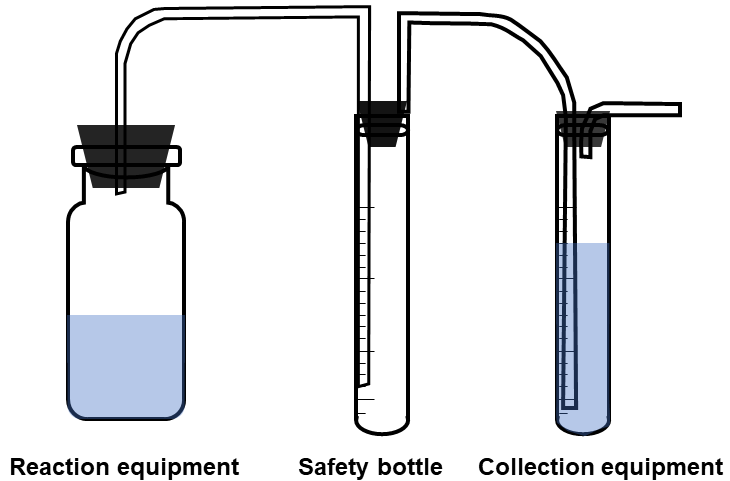


**Figure S5** Device for collecting gas generated by PHP pretreatment.


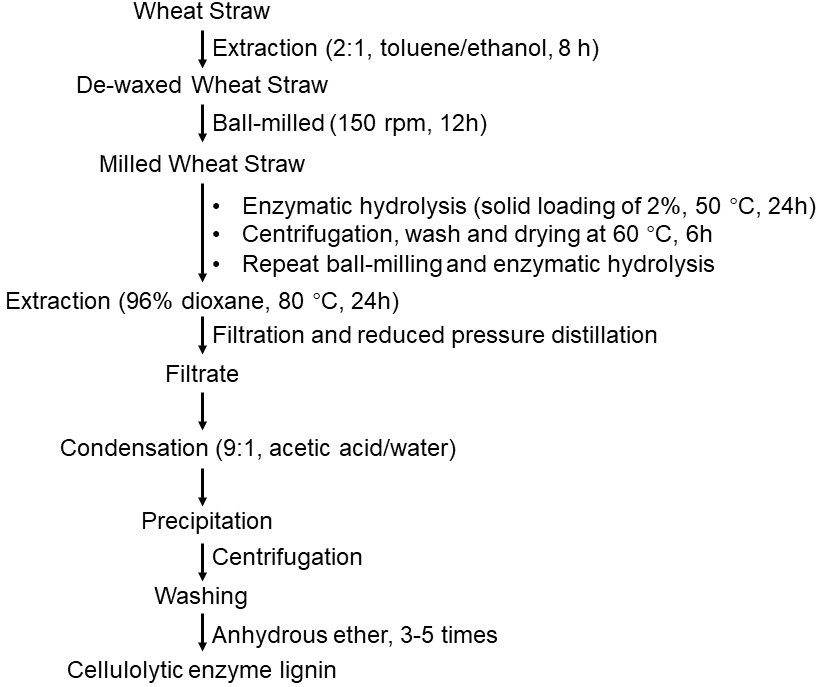


**Figure S6** Isolation procedure for cellulolytic enzyme lignin.
